# Graph analysis of diffusion tensor imaging-based connectome in young men with internet gaming disorder

**DOI:** 10.3389/fnins.2022.1090224

**Published:** 2023-01-30

**Authors:** Jiaolong Qin, Shuai Wang, Huangjing Ni, Ye Wu, Limin Chen, Shuaiyi Guo, Fuquan Zhang, Zhenhe Zhou, Lin Tian

**Affiliations:** ^1^PCA Lab, Key Lab of Intelligent Perception and Systems for High-Dimensional Information of Ministry of Education, School of Computer Science and Engineering, Nanjing University of Science and Technology, Nanjing, China; ^2^Jiangsu Key Lab of Image and Video Understanding for Social Security, School of Computer Science and Engineering, Nanjing University of Science and Technology, Nanjing, China; ^3^The Affiliated Mental Health Center of Jiangnan University, Wuxi Central Rehabilitation Hospital, Wuxi, China; ^4^School of Wuxi Medicine, Nanjing Medical University, Wuxi, China; ^5^School of Geographic and Biologic Information, Nanjing University of Posts and Telecommunications, Nanjing, China; ^6^The Affiliated Brain Hospital of Nanjing Medical University, Nanjing, China

**Keywords:** internet gaming disorder, cortico-limbic-striatal network, structural connectivity, DTI, connectome

## Abstract

Although recent evidence suggests that dysfunctional brain organization is associated with internet gaming disorder (IGD), the neuroanatomical alterations related to IGD remain unclear. In this diffusion tensor imaging (DTI) study, we aimed to examine alterations in white matter (WM) structural connectomes and their association with IGD characteristics in 47 young men with IGD and in 34 well-matched healthy controls. Two approaches [namely, network-based statistics (NBS) and graph theoretical measures] were applied to assess differences in the specific topological features of the networks and to identify the potential changes in the topological properties, respectively. Furthermore, we explored the association between the alterations and the severity of internet addiction. An NBS analysis revealed widespread alterations of the cortico-limbic-striatal structural connectivity networks in young people with IGD: (1) an increased subnet1 comprising the insula and the regions responsible for visual, auditory, and sensorimotor functions and (2) two decreased subnet2 and subnet3 comprising the insula, striatum, and limbic regions. Additional correlation analysis showed a significant positive association between the mean fractional anisotropy- (FA-) weighted connectivity strength of subnet1 and internet addiction test (IAT) scores in the IGD group. The present study extends our knowledge of the neuroanatomical correlates in IGD and highlights the role of the cortico-limbic-striatal network in understanding the neurobiological mechanisms underlying this disorder.

## Introduction

In China, internet-based behavioral impairments have been considered a serious social problem, and the so-called treatment programs have been the subject of debate for the last decade (Bax, 2015). In 2013, the American Psychiatric Association introduced Internet gaming disorder (IGD) in the third part of the Diagnostic and Statistical Manual of Mental Disorders, Fifth Edition (DSM-5) as a candidate diagnosis with requirements for further study (American Psychiatric Association [APA], 2013). Consequently, with the collection of more research evidence (Yao et al., 2017), “gaming disorder” was classified by the World Health Organization (WHO) as a new condition and was officially included in the 11th edition of the International Classification of Diseases (ICD-11) as an addictive behavior disorder (Gaebel et al., 2020). ICD-11 characterized this disorder as a persistent or recurring pattern of online or offline gaming behavior. However, considering that online gaming users in China have grown to approximately 540 million, or 57.4% of total internet users, in the first half of 2020,^1^ enormous attention has been paid to the phenomenon of clinical impairments or distress caused by maladaptive internet gaming (Yao et al., 2021).

The so-called IGD means excessive or poorly controlled preoccupations, urges, or behaviors regarding internet game playing that result in personal, familial, social, or occupational impairment for more than 12 months (American Psychiatric Association [APA], 2013). Moreover, IGD is often accompanied by depression and anxiety. Related research showed similarities in the neuropsychological processes between IGD and addictive substances (Grant et al., 2010). However, the pathological mechanisms underlying IGD remained elusive. Neuroimaging approaches were used to examine the underlying neurobiological mechanisms of IGD, and previous studies reported that IGD was associated with system-level alterations between the brain regions rather than functional impairment of isolated regions (Song et al., 2020). With the advent of connectomics, it is currently feasible to shift the view from an isolated regional perspective toward a system-level perspective (i.e., a network perspective) based on the integration of various forms of anatomical/functional data to assess the connectivity of networks in brain diseases including IGD (Bullmore and Sporns, 2009; Weinstein et al., 2017). A wide range of measures can be computed to assess the topological properties of the underlying brain networks (Rubinov and Sporns, 2010). To date, most studies addressing network alterations in IGD focused on functional networks derived from resting-state functional magnetic resonance imaging (rs-fMRI) (Lee et al., 2020; Song et al., 2020; Weinstein and Lejoyeux, 2020; Yan et al., 2021). These accumulated rs-fMRI studies on IGD demonstrated impaired interactions of functional brain networks involving the cortical-limbic-striatal circuitry that underlies reward processing, executive function, cognitive control, motor and sensory functions, and attention. Meanwhile, these studies provided novel evidence of aberrant core networks involving the central executive network, salience network, and default mode network (DMN) in this disorder (Weinstein et al., 2017; Chun et al., 2020; Weinstein and Lejoyeux, 2020).

An important question is whether the functional alterations observed across studies have a structural correlation. A study that focused on the covariance gray matter (GM) structural networks found higher GM volumes in DMN-related regions, which were associated with visuospatial attention and reward craving processing with increased severity of addiction to IGD (Chen et al., 2021). Using diffusion tensor imaging (DTI) data, previous studies reported increased fractional anisotropy (FA) values in the fasciculus linking reward circuitry, sensory, and motor control systems, which were related to the severity of internet addiction (Dong et al., 2012, 2018). Three studies established a white matter (WM) structural network to analyze its network metrics (namely, network controllability, and small-world topology) and reported that people with IGD had greater modular brain controllability and the shortest path length, as well as structural networks that shifted in the direction of random topology (Zhai et al., 2017; Park et al., 2018; Lei et al., 2020). However, very few studies examined alterations in the structural network in IGD using the connectomics method. To the best of our knowledge, no study has focused on WM structural network alterations in IGD using a network-based statistics (NBS) approach. In addition, this method can detect disrupted subnet patterns in whole brain networks.

The present study aime to identify differences in the WM structural connectome. To achieve our purpose, we included a fairly large sample of 47 young men with IGD and 34 well-matched male head circumferences (HCs) and applied the two approaches (namely, NBS and graph theoretical measures) to assess differences in the specific topological features of the networks and to identify potential changes in their topological properties. We hypothesized that the alterations in young people with IGD were mainly involved in the cortico-limbic-striatal network, which would likely be related to the severity of internet addiction.

## Materials and methods

### Participants

Given the higher prevalence of internet addiction in men vs. women in China (Li et al., 2013; Lau et al., 2017), only young men from local universities and the surrounding community were recruited *via* advertisements and word of mouth. Participants were preselected through an online questionnaire and telephone screening. In total, 47 young men who reported internet gaming as their primary online activity and met at least five of the nine DSM-5 criteria for IGD (American Psychiatric Association [APA], 2013) were screened. Subjects’ internet addictive behavior was assessed with the Chinese version of Internet Addiction Test (IAT) (Young, 1998). All young people with IGD were satisfied when their IAT score was more than the proposed cutoff score (i.e., ≥ 50) (Dong et al., 2015, 2018). Participants who met less than three of the nine criteria for IGD proposed by DSM-5 were preselected as having HC. Of these, 34 young people were determined to be HCs based on their IAT scores of less than 30. Additionally, participants’ current levels of depression and anxiety were assessed using the 24-item Hamilton Depression Scale (HAMD) and the 14-item Hamilton Anxiety Scale (HAMA) (Hamilton, 1959, 1967). All participants were right-handed as assessed with the Edinburgh Handedness Inventory (Oldfield, 1971). A brief, structured clinical interview tool, the Mini International Neuropsychiatric Interview, was used to screen for other psychiatric disorders. The exclusion criteria for selecting subjects were as follows: intracranial pathology, brain injury, neurological disorders, psychiatric disorders (except IGD), substance abuse, contraindications to MRI examinations, and excessive head motion. The demographic characteristics of young people with IGD and HC are summarized in Table 1.

**TABLE 1:** Demographic characteristics and clinical information of young people with internet gaming disorder (IGD) and healthy controls (HC).

Items	IGD (*N* = 47)	HC (*N* = 34)	*P*-value
Age (years)	20.60 ± 0.97	20.85 ± 1.79	0.41
14-HAMA	7.60 ± 5.10	3.81 ± 4.76▲	0.01*
24-HAMD	8.98 ± 6.40	4.50 ± 6.51▼	0.02*
Average gaming hours per week	23.26 ± 3.61	7.09 ± 3.33	<0.01*
IAT score	69.15 ± 8.11	21.62 ± 3.90	<0.01*
DMS-5 score	5.98 ± 1.01	1.76 ± 0.74	<0.01*

*Statistically significant.

^▲^Only 21 subjects measured HAMA.

^▼^Only 21 subjects measured HAMD.

Values are expressed as mean ± standard deviation.

IAT, internet addiction test; HAMA, Hamilton Anxiety Scale; HAMD, Hamilton Depression Scale; IGD, internet gaming disorder; HCs, healthy controls.

This study was approved by the Medical Ethics Committee of the Wuxi Mental Health Center, Nanjing Medical University, China. All subjects gave written informed consent before participating in the study.

### Imaging acquisitions and pre-processing

Magnetic resonance imaging scans were acquired with a 3.0-T Magnetom Trio Tim (Siemens Medical System, Erlangen, Germany) at the Department of Medical Imaging, The Affiliated Wuxi People’s Hospital of Nanjing Medical University. All participants obtained DTI data and high-resolution three-dimensional T1-weighted images. Foam pads were used to reduce head motion and noise from the scanner. T1-weighted images were acquired using a 3D-MPRAGE sequence with the following parameters: repetition time/echo time (TR/TE) = 2,300/2.98 ms, 160 sagittal slices, thickness = 1.2 mm, flip angle = 9°, matrix = 256 × 256, field of view (FOV) = 256 mm × 256 mm, and acquisition voxel size = 1 mm × 1 mm × 1.2 mm. DTI images were obtained with the following parameters: diffusion was measured along 64 non-collinear directions (*b*-value = 1,000 s/mm^2^), and one additional image without diffusion weighting (i.e., *b* = 0 s/mm^2^), TR/TE = 7,000 ms/92 ms, flip angle = 90°, FOV = 256 mm × 256 mm, matrix = 128 × 128, slice thickness/gap = 3/0 mm, and acquisition voxel size = 2 mm × 2 mm × 3 mm.

Image preprocessing was performed using the diffusion toolbox of the functional magnetic resonance imaging of the brain (FMRIB) software library (FSL).^2^ Visual and quantitative quality control of the DTI data was performed using a quality control tool in the FSL software.^3^ Moreover, from the analyses, individuals with diffusion images with apparent signal drops were checked, and images without this type of diffusion were found. Finally, images from all gradient directions were retained based on visual inspection of several patient data sets with an in-house tool, indicating that gradients should not be removed. Thus, all gradient directions were retained for the analysis. Pre-processing steps included eddy current and motion artifact correction, diffusion tensor estimation, and tractography. Corrections for eddy current distortions and head motion were performed by applying a rigid body transformation of each diffusion-weighted image to the b0 image. The *b*-matrix of each sample was then reoriented to provide a more accurate estimate of the tensor orientations. The diffusion tensor matrix was calculated according to the Stejskal and Tanner equation. Three eigenvalues and eigenvectors were obtained by diagonalizing the tensor matrix and then FA maps were computed. Each b0 image was registered in the Montreal Neurological Institute (MNI) space *via* the corresponding T1 image using Diffusionkit^4^ (Xie et al., 2016). The image registration of Diffusionkit is implemented by NiftyReg, which is open-source software for efficient registration of medical images and developed primarily by the Centre for Medical Image Computing at University College London. This transformation information was saved for later use. The diffusion images remained in native space.

Three-dimensional tract reconstruction was implemented using a diffusion toolkit.^5^ Whole brain tractography was obtained using the Fiber Assignment by Continuous Tracking algorithm (Mori et al., 1999), and propagation was terminated if a minimum angle threshold of 50° was violated or a voxel with FA below 0.2 was encountered.

### Construction of a structural network

**FIGURE 1:**
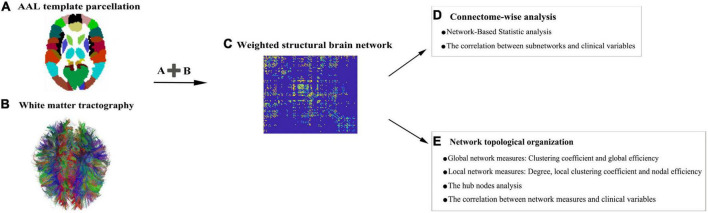
A flowchart for the construction of structural brain networks. **(A)** The 90 nodes of the brain network obtained from the automated anatomical labeling (AAL) atlas. **(B)** White matter (WM) fiber information prepared to check which pairs of nodes remain connected. **(C)** Region-based information extracted from the abovementioned image data. All pairwise connections between the nodes are calculated to generate a connection matrix. **(D,E)** A list of network analysis entries that are applied.

Figure 1 depicts the construction pipeline of a structural brain network. Specifically, the automated anatomical labeling (AAL) atlas^6^ (Tzourio-Mazoyer et al., 2002) with 90 regions (Supplementary Table 1) was employed as a node. Using the inverse of the transform information, the AAL atlas in the MNI space was registered into each subject’s native space. Edges were defined as interregional fibers between each pair of nodes and met the conditions: (1) at least two double-ended fibers passed through pairwise nodes and (2) the length of the passing fibers was greater than 10 mm. Here, the FA value was treated as the weight of a network connection. Specifically, the FA weight of each edge was calculated by averaging the FA values of all the fibers that made up this edge, and the FA value of each fiber was the mean of the FA values of all the voxels in this fiber track. A group threshold was applied to balance the influence of false-positive and false-negative reconstructions of fibers (de Reus and van den Heuvel, 2013). Edges that were present in at least 60% of all subjects were retained while others were zeroed. All subsequent analyses were conducted on this group threshold network.

### NBS analysis

Zalesky et al. (2010) proposed NBS, a non-parametric method, to eliminate the multiple-comparison problems encountered when conducting mass univariate significance tests. Statistical significance was detected to find the subnetwork, which consisted of specific subsets of nodes connected in the topological space. It is important to emphasize that no individual disconnection can be declared significant alone; only the disconnected subnetwork as a whole can be declared significant. We first used NBS to conduct an independent *t*-test analysis with age as a covariate. The general calculation procedures were as follows: (1) a primary threshold (*t*-value = 2.9, which was equal to setting *p* < 0.005 for the two-tailed test) was applied to a *t*-test, which was calculated for each edge to construct a set of suprathreshold connections. This identified all possible components (or subnetworks) mutually connected in a WM network at the primary threshold level. (2) The size of the actual remaining subnetwork *s* was determined. To estimate the significance of each subnetwork, the null distribution of the subnetwork size was empirically derived using a non-parametric permutation approach (5,000 permutations). For each permutation, all of the samples were shuffled randomly among the groups, and the *t*-test statistic was calculated independently for each edge. Afterward, the same threshold was applied to retain edges above this threshold, and the maximal subnetwork size was restored. (3) The corrected *p*-value was determined by calculating the proportion of the 5,000 permutations for which the maximum shuffled subnetwork was greater than *s*. A *p*-value of < 0.05 (corrected) was considered significant.

### Network measure analysis

For global network characteristics, we used the clustering coefficient and global efficiency. For local network measures, we computed three popular network metrics, namely, nodal degree, local clustering coefficient, and nodal efficiency. Additionally, betweenness centrality was used to define a hub node. Their formal mathematical definitions and meanings have been described in detail elsewhere (Rubinov and Sporns, 2010), and we also presented these descriptions in the Supplementary material. These measures were calculated on the WM network of each subject using the Brain Connectivity Toolbox.^7^ Before making between-group comparisons, the interaction between age and network metrics was regressed. Between-group significances of network metrics were determined using an independent two-sample *t*-test (two-tailed) with the Bonferroni’s correction (*p* < 0.05/90).

### Pearson’s correlation analysis

Pearson’s correlation analysis was used to examine the relationship between network measures and clinical variables (namely, IAT, average gaming hours per week, and DSM-5 score). In addition, we also investigated the association between the mean FA-weighted structural connectivity strength of each subnetwork and the clinical variables.

## Results

### Differences in structural connectivity patterns

**FIGURE 2:**
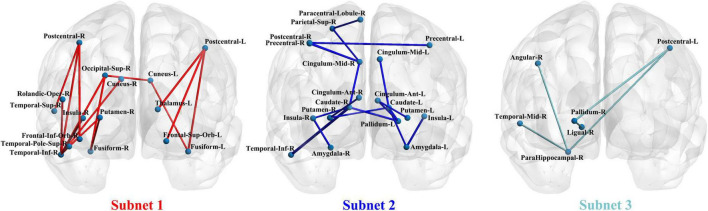
The three subnetwork results from the network-based statistics (NBS) analysis in a coronal view.

Network-based statistical analysis identified three disconnected structural subnetworks in the IGD group (Table 2 and Figure 2). Compared with HCs, young people with IGD showed a significant increase in connectivity strength in subnet1 with 16 edges and involving the bilateral orbitofrontal regions and visual regions (namely, the bilateral cuneus and fusiform gyrus, and the right superior occipital gyrus), auditory regions (i.e., the right temporal pole), and sensorimotor regions (namely, the bilateral post-central gyrus and the right inferior temporal gyrus) (corrected *p* < 0.0012). Furthermore, in young people with IGD, network disconnections comprising two subnetworks had significantly fewer connections. Subnet2 comprised 18 edges and was mainly within and between the limbic (namely, the bilateral amygdala, and the median cingulate) and the striatum (namely, the bilateral caudate, the putamen, and the left pallidum), the bilateral insula, and several areas responsible for visual/auditory and sensorimotor functions (namely, the bilateral precentral gyrus, the left post-central gyrus, the right superior parietal gyrus, the right paracentral lobule, and the right inferior temporal gyrus) (corrected *p* < 0.0006). Subnet3 included five edges, was centered with the right parahippocampal gyrus and the pallidum, and connected the regions of the right hemisphere including the post-central gyrus, the angular gyrus, the lingual gyrus, and the middle temporal gyrus (corrected *p* < 0.018).

**TABLE 2:** Subnetwork with a significant between-group difference based on the network-based statistics (NBS) analysis.

Network edges	*T*- value (*P*-value)	Network edges	*T*-value (*P*-value)
**IGD increase, subnet1**		**IGD decrease, subnet2 (continue)**	
Cuneus_L – Occipital_Sup_R	3.04 (*p* < 0.005)	Cingulum_Mid_R – Postcentral_R	3.00 (*p* < 0.005)
Cuneus_L – Fusiform_L	2.94 (*p* < 0.005)	Cingulum_Mid_R – Parietal_Sup_R	2.97 (*p* < 0.005)
Occipital_Sup_R – Fusiform_R	3.35 (*p* < 0.005)	Parietal_Sup_R – Paracentral_Lobule_R	3.11 (*p* < 0.005)
Frontal_Sup_Orb_L – Postcentral_L	3.51 (*p* < 0.005)	Cingulum_Mid_L – Caudate_L	3.33 (*p* < 0.005)
Fusiform_L – Postcentral_L	3.37 (*p* < 0.005)	Amygdala_L – Caudate_L	3.02 (*p* < 0.005)
Frontal_Inf_Orb_R – Postcentral_R	3.43 (*p* < 0.005)	Insula_R – Caudate_R	2.94 (*p* < 0.005)
Fusiform_R – Putamen_R	2.98 (*p* < 0.005)	Cingulum_Mid_R – Caudate_R	3.34 (*p* < 0.005)
Postcentral_L – Thalamus_L	3.54 (*p* < 0.005)	Cingulum_Ant_L – Putamen_L	2.99 (*p* < 0.005)
Postcentral_R –Temporal_Sup_R	3.13 (*p* < 0.005)	Cingulum_Ant_R – Putamen_R	3.00 (*p* < 0.005)
Putamen_R – TPOsup.R	3.03 (*p* < 0.005)	Caudate_L – Putamen_R	2.99 (*p* < 0.005)
Rolandic_Oper_R – Temporal_Inf_R	3.30 (*p* < 0.005)	Cingulum_Ant_L – Pallidum_L	2.99 (*p* < 0.005)
Insula_R – Temporal_Inf_R	3.62 (*p* < 0.005)	Caudate_L – Pallidum_L	3.12 (*p* < 0.005)
Cuneus_R – Temporal_Inf_R	3.19 (*p* < 0.005)	Caudate_R – Pallidum_L	3.51 (*p* < 0.005)
Occipital_Sup_R – Temporal_Inf_R	3.37 (*p* < 0.005)	Caudate_R – Temporal_Inf_R	4.55 (*p* < 0.005)
Postcentral_R – Temporal_Inf_R	3.38 (*p* < 0.005)	**IGD decrease, subnet3**	
Temporal_Pole_Sup_R – Temporal_Inf_R	2.91 (*p* < 0.005)	ParaHippocampal_R – Postcentral_L	2.98 (*p* < 0.005)
**IGD decrease, subnet2**		ParaHippocampal_R – Angular_R	2.92 (*p* < 0.005)
Precentral_R – Cingulum_Mid_R	3.61 (*p* < 0.005)	Lingual_R – Pallidum_R	3.05 (*p* < 0.005)
Insula_L – Amygdala_L	3.62 (*p* < 0.005)	Postcentral_L – Pallidum_R	3.37 (*p* < 0.005)
Insula_R – Amygdala_R	3.35 (*p* < 0.005)	ParaHippocampal_R – Temporal_Mid_R	3.17 (*p* < 0.005)
Precentral_L – Postcentral_R	3.24 (*p* < 0.005)		

### Network measures

Significant group effects on network clustering (*t*-value = 2.79, *p* = 0.0007) and global efficiency (*t*-value = 3.70, *p* = 0.0004) were observed in the analyses between young people with IGD and HC. The bilateral orbital frontal parts include two regions both left and right orbital frontal regions. Analogously, the bilateral supplementary motor areas are as the same, the left dorsolateral frontal part, and the right medial frontal part), one temporal region (the right superior temporal gyrus), one parietal region (the right paracentral lobule), three cingulum regions (namely, the bilateral median, and the right anterior cingulate), the right insula and subcortical nuclei (namely, the right parahippocampal gyrus, and the left putamen) (Tables 3–5). Specifically, compared with HC, young people with IGD displayed a significantly higher nodal efficiency and nodal degree in the right orbital part of the inferior frontal gyrus and left supplementary motor area. Moreover, young people with IGD also showed a significant increase in the local clustering coefficient in the right insula and a higher nodal degree in the right superior temporal gyrus.

**TABLE 3:** Differences in local clustering coefficient between young people with IGD and HC.

Metric	Region	IGD (mean ± std)	NC (mean ± std)	*T*-value (*P*-value)
C*_*i*_*	Insula_R	0.012 ± 0.020	−0.017 ± 0.045	3.92 (1.89e-04)
C*_*i*_*	Cingulum_Ant_R	0.040 ± 0.080	−0.056 ± 0.134	4.03 (1.29 e-04)
C*_*i*_*	Paracentral_Lobule_R	0.025 ± 0.044	−0.034 ± 0.088	3.94 (1.73 e-04)

C*_i_*, local clustering coefficiency.

**TABLE 4:** Differences in the nodal degree between young people with IGD and HC.

Metric	Region	IGD (mean ± std)	NC (mean ± std)	*T*-value (*P*-value)
D*_*i*_*	Frontal_Inf_Orb_L	0.609 ± 1.067	−0.843 ± 2.457	3.61 (5.30e-04)
D*_*i*_*	Frontal_Inf_Orb_R	0.633 ± 1.187	−0.875 ± 2.351	3.79 (2.97e-04)
D*_*i*_*	Supp_Motor_Area_L	0.377 ± 0.731	−0.521 ± 1.223	4.12 (9.24e-05)
D*_*i*_*	Cingulum_Mid_L	0.507 ± 1.153	−0.701 ± 1.769	3.72 (3.74e-04)
D*_*i*_*	Cingulum_Mid_R	0.394 ± 0.886	−0.544 ± 1.295	3.87 (2.20e-04)
D*_*i*_*	Temporal_Sup_R	0.301 ± 0.621	−0.416 ± 0.990	4.00 (1.40e-04)

D*_i_*, nodal degree.

**TABLE 5:** Differences in nodal efficiency between young people with IGD and HC.

Metric	Region	IGD (mean ± std)	NC (mean ± std)	*T*-value (*P*-value)
E*_*i*_*	Frontal_Sup_L	0.022 ± 0.047	−0.031 ± 0.075	3.88 (2.12e-04)
E*_*i*_*	Frontal_Inf_Orb_R	0.031 ± 0.054	−0.043 ± 0.124	3.68 (4.24e-04)
E*_*i*_*	Supp_Motor_Area_L	0.020 ± 0.047	−0.028 ± 0.063	4.00 (1.41e-04)
E*_*i*_*	Supp_Motor_Area_R	0.023 ± 0.056	−0.031 ± 0.072	3.78 (3.01e-04)
E*_*i*_*	Frontal_Sup_Medial_R	0.018 ± 0.041	−0.024 ± 0.061	3.72 (3.74e-04)
E*_*i*_*	Cingulum_Mid_L	0.028 ± 0.052	−0.039 ± 0.109	3.71 (3.90e-04)
E*_*i*_*	ParaHippocampal_R	0.026 ± 0.049	−0.037 ± 0.094	3.90 (2.01e-04)
E*_*i*_*	Paracentral_Lobule_L	0.032 ± 0.051	−0.045 ± 0.108	4.28 (5.22e-05)
E*_*i*_*	Putamen_L	0.018 ± 0.041	−0.025 ± 0.068	3.61 (5.32e-04)

E*_i_*, nodal efficiency.

**FIGURE 3:**
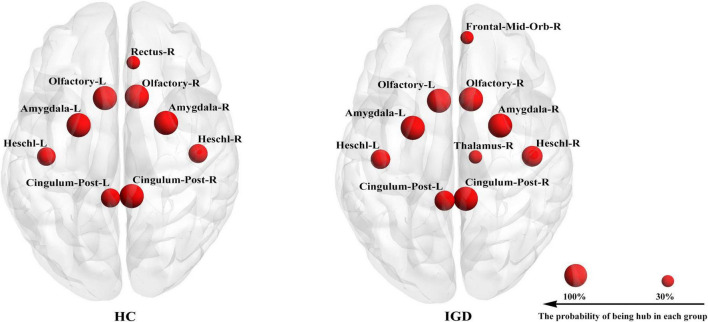
Each group has its own hub distribution. It showed the hub with a probability of being a hub greater than 30%.

The hub detection result revealed the similarities in the hub distribution of young people with IGD and HC (Figure 3). In hub distribution, 8 nodes (namely, the bilateral precuneus, the bilateral amygdala, the bilateral pallidum, and the bilateral Heschl gyrus) were working as hubs with 100% probability in both groups.

### The correlation results

**FIGURE 4:**
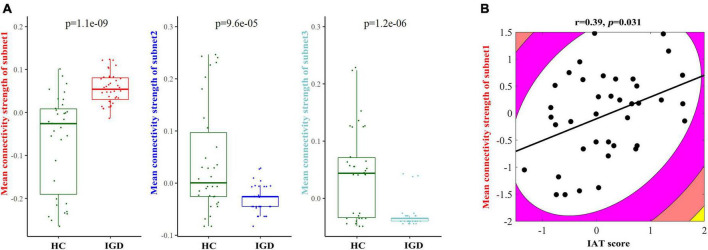
**(A)** Between-group comparison results of the mean fractional anisotropy- (FA–) weighted structural connectivity strength in each subnetwork between young people with internet gaming disorder (IGD) and healthy controls (HC). **(B)** An illustration of the correlation result between the mean FA-weighted structural connectivity strength of subnet1 and the IAT score in the IGD group.

We also compared the mean FA values of the edges of each subnetwork between the IGD and HC groups, and their comparison results showed the existence of significant between-group differences (Figure 4A). Because most of the patients with non-existing edges were in the decreasing subnet2 and subnet3, we only did the correlation analysis in the increasing subnet1. The mean FA-weighted connectivity strength of subnet1 was positively related to IAT scores (*r* = 0.39, *p* = 0.031) (Figure 4B). In addition, a positive correlation was found between the DSM-5 scores and the degree of the right superior temporal gyrus (*r* = 0.37, *p* = 0.033).

## Discussion

**FIGURE 5:**
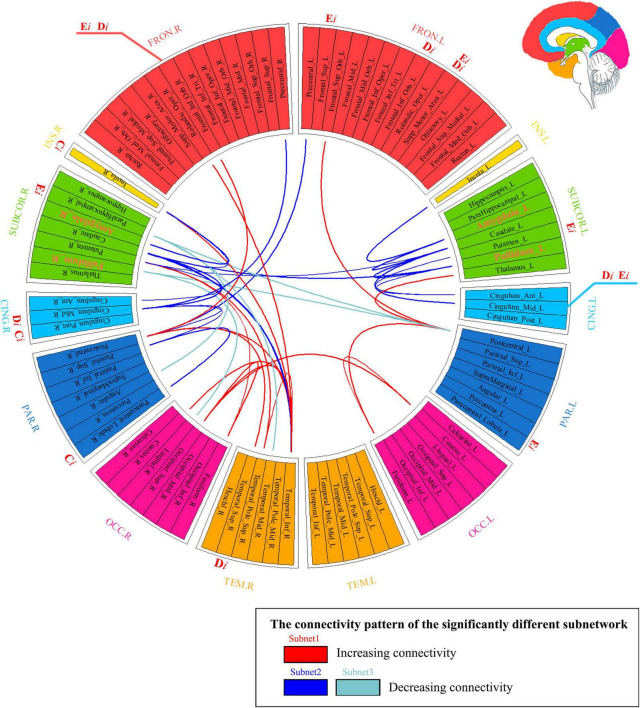
Summarizing the results of network analysis. It represented three subnetwork results (i.e., subnetworks 1–3) whose connections are marked with different colors in the connectogram. If a nodal measure showed the existence of a significant between-group difference, the location of the outer ring of the circle corresponding to this node was labeled as the nodal measure’s name (namely *D*_*i*_, *E*_*i*_, and *C*_*i*_). If the nodal measure’s name was colored with red, it meant that the corresponding value increased. Hubs’ name was marked with orange color and with a larger font size in the ring. FRON, frontal cortex; INS, insula; SUBCOR, subcortical region; CING, cingulum; PAR, parietal cortex; OCC, occipital cortex; TEM, temporal cortex; L, left hemisphere; R, right hemisphere.

Our findings indicated the following: (1) There were three abnormal subnets between young people with IGD and HC. Subnet1 showed an increase in the mean FA-weighted connectivity strength among the orbitofrontal area, the insula, and the visual, auditory, and sensorimotor regions, while the other two subnets (namely, subnet2, and subnet3) presented a decrease in the mean FA-weighted structural connectivity strength within and between the limbus, the striatum, and the insula. (2) Relative to HCs, young people with IGD showed an increase in network clustering and global efficiency. In addition, young people with IGD displayed significantly higher local clustering coefficient, nodal degree, and nodal efficiency in the orbital, dorsolateral, and medial frontal areas; the right parahippocampal gyrus; the left putamen; the right insula; the right superior temporal gyrus; the right paracentral lobule; and the cingulum. (3) The correlation results indicated an enhancement in the mean FA-weighted structural connectivity strength of subnet1 was positively related to the severity of IAT. Moreover, the right superior temporal gyrus, one of the nodes in subnet1, showed that its highest degree value was positively correlated with DSM-5 scores. Figure 5 summarizes all these abovementioned results. Our results suggest that abnormal connections occur in the cortico-striatal-limbic circuitry that may be the result of excessive internet gaming.

### Disorder-related distinctions of subnetworks in WM networks

As the NBS results showed an increase in the mean FA-weighted structural connectivity strength of subnet1, which included three brain regions (namely, the right orbital part of the inferior frontal gyrus, the right putamen, and the right insula) responsible for reward activity through the sensorimotor (namely, the right post-central gyrus, and the right inferior temporal gyrus) and visual (namely, the right superior occipital gyrus, and the right fusiform gyrus) areas to link together. The insula plays an important role in maintaining homeostasis through the integration of internal and external stimuli to guide behavior toward or away from the same stimuli (Gogolla, 2017). Specifically, the insula is associated with an increased tendency to engage in disgusting behaviors and an impaired ability to recognize disgust in others (Woolley et al., 2015). Accumulated evidence demonstrated the role of the insula in addictive behavior and suggested its promise as a target for the treatment of addiction (Ibrahim et al., 2019). The orbitofrontal area is the key brain area in the representation of reward or non-reward value, and is capable of controlling and correcting reward-related and punishment-related behaviors (Ibrahim et al., 2019). The putamen is involved in the processing of primary rewards and visual events in a complex task, which may play an important role in reinforcement learning through stimulus-reward association (Vicente et al., 2012). A growing body of evidence suggested that IGD was associated with alterations in brain regions responsible for reward processing and sensorimotor function (Weinstein et al., 2017; Dong et al., 2018; Weinstein and Lejoyeux, 2020). Our findings are in line with these previous studies. Furthermore, in the IGD group, the right insula showed an enhanced local clustering coefficient and was linked to the right inferior temporal gyrus, which was a key component of subnet1. An enhanced local clustering coefficient of the right insula suggested that its short-distance connections were increased. In subnet1, the right inferior temporal gyrus was a central node with a higher degree, and its majority edges were linked to areas responsible for visual and sensorimotor functions (namely, the right cuneus, the right superior occipital gyrus, the right post-central gyrus, and the right temporal pole of the superior temporal gyrus). These results may indicate that increased short connections of the right insula come mainly from those regions associated with visual and sensorimotor functions, which may cause the insula to enhance its response to external stimuli. Thus, we speculate that the excessive use of the internet for gaming strengthens the connections among those regions responsible for visual, sensorimotor, and reward functions, which may increase individuals’ feelings of game experience (i.e., subjective pleasure) and may aggravate their addictive behaviors.

In addition, a decrease in the mean FA-weighted structural connectivity strength of subnet2 and subnet3 was shown, and most of these altered connections were located mainly within the limbic areas (namely, the amygdala, the parahippocampal gyrus, the anterior cingulate gyrus, and the median cingulate gyrus), the striatum areas (namely, the caudate, the putamen, and the pallidum), and the bilateral insula. The striatum is thought to play an important role in the pathophysiology of IGD, and prior studies identified a dopamine-driven striatal function as a core candidate for promoting addictive behavior (Weinstein, 2010; Kuss and Griffiths, 2012; Tian et al., 2014; Weinstein et al., 2017). Recently, related brain imaging studies implicated the important role of the dysfunctional limbic system (especially the amygdala, the parahippocampal gyrus, and the cingulate gyrus) in the neuropathological mechanism of IGD (Weinstein, 2010; Kuss and Griffiths, 2012; Tian et al., 2014; Weinstein et al., 2017). The connections between the bilateral amygdala and the insula constituted an important part of subnet2. Berret et al. (2019) applied optogenetic approaches in the animal model and corroborated that the insula routed aversive somatosensory information to the amygdala and contributed to elaborate its negative valence, thus suppressing an essential drive to learn about such harmful information. The insula along with the amygdala is necessary for the retrieval of threat memories. Furthermore, Baur et al. (2013) analyzed resting-state functional and structural connectivity within the amygdala-insula in healthy subjects and demonstrated that this connectivity represented the index state and trait anxiety. Ko et al. (2015) examined the amygdala-centered network in IGD on the basis of rs-fMRI and found altered connectivity of the bilateral amygdala and the insula in adults with IGD than in controls. Hence, these decreased structural subnets, to a certain extent, may affect reward processing, emotion processing and regulation, and cognitive control. Moreover, the involvement of the connectivity between the bilateral amygdala and the insula in these subnets may give a potential cue that young people with IGD underestimate the negative valence of excessive play and neglect the harmfulness from such behavior.

Taken together, these findings provide tentative evidence for enhanced structural connectivity among the insula, the sensorimotor, visual, and reward-related regions, and decreased structural connectivity in the insula, limbic, and striatal regions. Among these, the insula holds quite an important position in these findings, which suggest that, on the one hand, excessive play of internet games intensifies sensory stimuli and improves subjective pleasure and, on the other hand, individuals with IGD weaken their awareness of the risk of indulging in internet games. All these results may provide a potential explanation for why young people indulge in playing internet games and highlight the role of the insula in the pathological mechanism of IGD.

### An increase in the mean FA-weighted structural connectivity strength of subnet1 was related to the severity of internet addiction

The result showed that the mean FA-weighted structural connectivity strength of subnet1 was significantly positively related to the IAT score in young people with IGD. The IAT score can reflect the severity of the gaming addiction. This result implies that the higher the connectivity strength of subnet1, the more severe the impairment in IGD. As mentioned above, subnet1 exhibited an enhancement in the mean FA-weighted structural connectivity strength in young people with IGD and was involved in the insula and the frontal, visual, auditory, and sensorimotor regions. These areas are responsible for receiving external sensory stimuli and processing reward-related functions. Previous cross-sectional and longitudinal studies demonstrated that training can induce changes in WM structure. Bengtsson et al. (2005) found that the amount of piano practice in childhood enhanced FA values within related motor tracts. Scholz et al. (2009) designed a longitudinal study of individuals learning a new visuomotor skill, juggling, and reported an increase in the FA value of the intraparietal sulcus in the juggling group. An individual with excessive gaming use over a long period of time can induce experience-dependent changes in WM within regions responsible for visual, auditory, and sensorimotor processing. For example, Jeong et al. (2016) reported that men with IGD increased FA values within several fasciculi such as the forceps minor, the right corticospinal tract, the right inferior longitudinal fasciculus, and the right inferior fronto-occipital fasciculus, and also found a positive correlation of the FA values of these fasciculi with the duration of illness in IGD. These fasciculi play an important role in visual, auditory, motor, and working memory functions. Although WM in men with IGD was measured using different analysis methods, our findings showed three similarities (such as predominance in the right hemisphere, an increased FA value, and an impact on the WM bundles responsible for visual/auditory/sensorimotor function) with these of Jeong et al. (2016). For example, (1) the subnet1 result predominated within the right hemisphere; (2) subnet1 increased the mean FA-weighted structural connectivity value; and (3) most nodes in subnet1 were responsible for receiving the external stimuli. We speculate that enhanced WM structural connectivity within subnet1 may arise secondary to long-term internet gaming addiction and may affect the processing of visual/auditory/sensorimotor and reward functions.

### Limitations and conclusion

Several limitations of the present study should be noted. First, the current study only recruited young men. Some previous neuroimaging studies revealed gender-related differences in IGD (Jeong et al., 2016), and men were more vulnerable to IGD than women (Borgonovi, 2016). Some scholars urged considering the importance of gender in understanding IGD (King and Potenza, 2020). Our study excluded the effect of gender on the findings. However, these findings should be considered specific to young men with IGD. Future studies need to verify these results in women and in those with other occupations. In addition, we have not obtained the HAMA and HAMD scores of all HC subjects, because of the lack of willingness to tests by some of them. However, in the preliminary HAMA/HAMD data, patients with IGD showed a significantly higher score than those with HCs. Considering psychological variables, depression and anxiety are more strongly associated with the development of IGD. Higher depression and anxiety might be representative indicators of the problems of individuals with IGD. If all HC subjects without HAMA/HAMD scores were removed and additional analyses were performed with HAMA/HAMD as a covariate, it still would not be possible to explain which factors (namely, HAMA/HAMD and a change in the number of HC subjects) affect the results and how much influence these factors have on the results, respectively. Due to this objective consideration, we had not included both variables as covariates in our primary data analyses. Further studies that explicitly recruit individuals with IGD and also low levels of anxiety and depression are needed to disentangle the effects of both variables on the structural connectivity networks. Finally, a cross-sectional study can never confirm a causal role for experience on the WM structure in brains, due to the possibility that common genetic factors influence both the WM structure and the propensity to train. A longitudinal study is needed to further corroborate whether altered subnets are a consequence of excessive use of internet games.

In conclusion, the present study is the first to assess WM structural network alterations in IGD using an NBS approach from a perspective of connectomics. We observed a widespread alteration of cortico-limbic-striatal structural connectivity networks, including an increased subnet1 (mainly involving the insula and regions responsible for visual, auditory, and sensorimotor functions) and decreased two subnet2 and subnet3 (mainly in the insula, striatum, and limbic regions). Moreover, the mean FA-weighted structural connectivity of subnet1 showed a significant positive relationship with the severity of internet addiction. In particular, the insula appeared in both increased and decreased subnets, playing an important position in these findings, highlighting its role in understanding the neurobiological mechanisms underlying IGD and in developing effective treatment strategies for this disorder.

## Data availability statement

The raw data supporting the conclusions of this article will be made available by the authors, without undue reservation.

## Ethics statement

This study was approved by the Medical Ethics Committee of Wuxi Mental Health Center, Nanjing Medical University, China. The patients/participants provided their written informed consent to participate in this study.

## Author contributions

JQ was responsible for analyzing data and writing the manuscript. SW was responsible for data collection and writing the manuscript. LT was responsible for the study design and revising the manuscript. HN and YW were responsible for analyzing data and revising the manuscript. LC, FZ, and ZZ were responsible for data collection and recruiting for patients. All authors have critically reviewed the content and approved the final version submitted for publication.

## Footnotes

1
http://www.cnnic.net.cn/

2
http://fsl.fmrib.ox.ac.uk/fsl/fslwiki/

3
https://fsl.fmrib.ox.ac.uk/fsl/fslwiki/eddyqc/UsersGuide

4
https://www.nitrc.org/projects/diffusionkit

5
http://www.trackvis.org

6
http://www.gin.cnrs.fr/en/tools/aal-aal2/

7
http://www.nitrc.org/projects/bct/
