# Supplementary material for: Graph analysis of diffusion tensor imaging-based connectome in young men with internet gaming disorder
Source: Front Neurosci. 2023 Jan 30;16:1090224. doi: 10.3389/fnins.2022.1090224 (PMC9926964; doi:10.3389/fnins.2022.1090224)
Supplement: Supplementary file 1 [file Data_Sheet_1.doc]

**Supplementary materials**

**Table S1.** Cortical regions of interest defined in the current study

| Index | Regions | Abbreviation | Index | Regions | Abbreviation |
| --- | --- | --- | --- | --- | --- |
| 1,2 | Precental gyrus | Precentral-L/R | 47,48 | Lingual gyrus | Lingual-L/R |
| 3,4 | Superior frontal gyrus, dorsolateral | Frontal-Sup-L/R | 49,50 | Superior occipital gyrus | Occipital-Sup-L/R |
| 5,6 | Superior frontal gyrus, orbital part | Frontal-Sup-Orb-L/R | 51,52 | Middle occipital gyrus | Occipital-Mid-L/R |
| 7,8 | Middle frontal gyrus | Frontal-Mid-L/R | 53,54 | Inferior occipital gyrus | Occipital-Inf-L/R |
| 9, 10 | Middle frontal gyrus, orbital part | Frontal-Mid-Orb-L/R | 55,56 | Fusiform gyrus | Fusiform-L/R |
| 11,12 | Inferior frontal gyrus, opercular part | Frontal-Inf-Oper-L/R | 57,58 | Postcentral gyrus | Postcentral-L/R |
| 13,14 | Inferior frontal gyrus, triangular part | Frontal-Inf-Tri-L/R | 59,60 | Superior parietal gyrus | Parietal-Sup-L/R |
| 15,16 | Inferior frontal gyrus, orbital part | Frontal-Inf-Orb-L/R | 61,62 | Inferior parietal, but supramarginal and angular gyri | Parietal-Inf-L/R |
| 17,18 | Rolandic operculum | Rolandic-Oper-L/R | 63,64 | Supramarginal gyrus | SupraMarginal-L/R |
| 19,20 | Supplementary motor area | Supp-Motor-Area-L/R | 65,66 | Angular gyrus | Angular-L/R |
| 21,22 | Olfactory cortex | Olfactory-L/R | 67,68 | Precuneus | Precuneus-L/R |
| 23,24 | Superior frontal gyrus, medial | Frontal-Sup-Medial-L/R | 69,70 | Paracentral lobule | Paracentral-Lobule-L/R |
| 25,26 | Superior frontal gyrus, medial orbital | Frontal-Mid-Orb-L/R | 71,72 | Caudate nucleus | Caudate-L/R |
| 27,28 | Gyrus rectus | Rectus-L/R | 73,74 | Lenticular nucleus, putamen | Putamen-L/R |
| 29,30 | Insula | Insula-L/R | 75,76 | Lenticular nucleus, pallidum | Pallidum-L/R |
| 31,32 | Anterior cingulate and paracingulate gyri | Cingulum-Ant-L/R | 77,78 | Thalamus | Thalamus-L/R |
| 33,34 | Median cingulate and paracingulate gyri | Cingulum-Mid-L/R | 79,80 | Heschl gyrus | Heschl-L/R |
| 35,36 | Posterior cingulate gyrus | Cingulum-Post-L/R | 81,82 | Superior temporal gyrus | Temporal-Sup-L/R |
| 37,38 | Hippocampus | Hippocampus-L/R | 83,84 | Temporal pole: superior temporal gyrus | Temporal-Pole-Sup-L/R |
| 39,40 | Parahippocampal gyrus | ParaHippocampal-L/R | 85,86 | Middle temporal gyrus | Temporal-Mid-L/R |
| 41,42 | Amygdala | Amygdala-L/R | 87,88 | Temporal pole: middle temporal gyrus | Temporal-Pole-Mid-L/R |
| 43,44 | Calcarine fissure and surrounding cortex | Calcarine-L/R | 89,90 | Inferior temporal gyrus | Temporal-Inf-L/R |
| 45,46 | cuneus | Cuneus-L/R |  |  |  |

Note: The regions are listed based on the template obtained from the AAL atlas. L, left hemisphere; R, right hemisphere.

**Network measure analysis**

To describe the topological organization of white matter structural networks, two graph measures were included here, as below: nodal strength and nodal efficiency. We used betweenness centrality to define hubs and this metric was introduced here. Regarding a review on the uses and interpretations of the measures, see (Rubinov et al.,2010) and the following characterizations.

*Nodal strength*

In a weighted graph G with n nodes, the nodal strength s*_i_* for node *i* was defined as the sum of the weights of direct connecitons of node *i*:

$$s_{i}=\sum_{j\in N} W_{ij}$$

[1]

where N was the set of all nodes in the graph G, and W*ij* was the weight between node *i* and node *j* in the graph. Obviously, the network strength S of network G was computed as the average of s*_i_* for all nodes within set N. Formmaly:

$$S=\frac{1}{n}\sum_{i\in N} s_{i}$$

[2]

where n was the number of nodes.

*Nodal efficiency*

In a weighted graph G with n nodes, the nodal efficiency e*i* of a node *i* was calculated as the mean of the inverse of the distances of all nodes that directly connected node *i* except the node per se. Formally:

$$e_{i}=\frac{1}{n-1}\sum_{j\in,j\neq i} \frac{1}{d_{ij}}$$

[3]

where d*ij* was the shortest path length between node *i* and *j* in G, and N was the set of all nodes in the graph. This metric quantified the importance of the nodes for communication within the network. In turn, the global efficiency of a network G with *n* nodes was calculated as:

$$E_{glob}(G)=\frac{1}{n}\sum_{i\in N} e_{i}$$

[4]

*Betweenness centrality and hub definition*

In a weighted graph G with N nodes, the betweenness centrality b*i* of a node *i* represented a central node that played a key role in control over the information transfer within the G, which was computed as the fraction of shortest paths that passed through node *i* between other nodes. Formally:

$$b_{i}=\frac{1}{(n-1)(n-2)}\sum_{h,j\in N,h\neq i,j,i\neq j} \frac{sp_{hj}(i)}{sp_{hj}}$$

[6]

where sp*_hj_*(*i*) was the number of shortest path between node *h* and node *j* passing through node *i*, and N was the set of all nodes in the G.

We used betweenness centrality metric to define the hub nodes as follows. For identification of hubs in WM networks, we computed the normalized betweenness centrality as $h_{i}=b_{i}/\left\langle b_{i}\right\rangle$, where $\left\langle b_{i}\right\rangle$ was the mean nodal betweenness centrality of each FA-weighted WM network. A node was classified as a hub, depending on whether its $h_{i}$ value was 1.5 times larger than the $\left\langle b_{i}\right\rangle$ of this network. Thus, each WM network had defined its own hubs.

**References:**

Rubinov, M., Sporns, O. (2010). Complex network measures of brain connectivity: uses and interpretations. Neuroimage. 52(3), 1059-1069
